# The use of exhaled air analysis in discriminating interstitial lung diseases: a pilot study

**DOI:** 10.1186/s12931-021-01923-5

**Published:** 2022-01-20

**Authors:** L. Plantier, A. Smolinska, R. Fijten, M. Flamant, J. Dallinga, J. J. Mercadier, D. Pachen, M. P. d’Ortho, F. J. van Schooten, B. Crestani, A. W. Boots

**Affiliations:** 1 Department of Pulmonology and Lung Function Testing, CHRU, Tours, France; 2 Université de Tours, Tours, France; 3 Centre d’Etude des Pathologies Respiratoires, INSERM UMR1100, Tours, France; 4 Department of Pharmacology and Toxicology, NUTRIM School of Nutrition and Translational Research in Metabolism, Maastricht University, Maastricht, The Netherlands; 5 Service de Physiologie – Explorations Fonctionnelle, Assistance Publique-Hôpitaux de Paris, Hôpital Bichat, Paris, France; 6 Service de Pneumologie A, DHU FIRE, Assistance Publique-Hôpitaux de Paris, Hôpital Bichat, Paris, France; 7 Université Paris Diderot, PRES Sorbonne Paris Cité, Paris, France; 8 INSERM UMR1152, Labex Inflamex, Paris, France; 9 Department of Radiation Oncology (Maastro) GROW School for Oncology and Developmental Biology, Maastricht University Medical Centre, 6229 ET Maastricht, The Netherlands; 10 Université de Paris, INSERM UMR 1141, NeuroDiderot, France

**Keywords:** Volatile organic compound (VOC), Idiopathic pulmonary fibrosis (IPF), Connective tissue disease associated-ILD (CTD-ILD), Diagnostic profiles, Gas chromatography*–time of flight*–mass spectrometry (GC-*tof*–MS)

## Abstract

**Background:**

Fibrotic Interstitial lung diseases (ILD) are a heterogeneous group of chronic lung diseases characterized by diverse degrees of lung inflammation and remodeling. They include idiopathic ILD such as idiopathic pulmonary fibrosis (IPF), and ILD secondary to chronic inflammatory diseases such as connective tissue disease (CTD). Precise differential diagnosis of ILD is critical since anti-inflammatory and immunosuppressive drugs, which are beneficial in inflammatory ILD, are detrimental in IPF. However, differential diagnosis of ILD is still difficult and often requires an invasive lung biopsy. The primary aim of this study is to identify volatile organic compounds (VOCs) patterns in exhaled air to non-invasively discriminate IPF and CTD-ILD. As secondary aim, the association between the IPF and CTD-ILD discriminating VOC patterns and functional impairment is investigated.

**Methods:**

Fifty-three IPF patients, 53 CTD-ILD patients and 51 controls donated exhaled air, which was analyzed for its VOC content using gas chromatograph- *time of flight*- mass spectrometry.

**Results:**

By applying multivariate analysis, a discriminative profile of 34 VOCs was observed to discriminate between IPF patients and healthy controls whereas 11 VOCs were able to distinguish between CTD-ILD patients and healthy controls. The separation between IPF and CTD-ILD could be made using 16 discriminating VOCs, that also displayed a significant correlation with total lung capacity and the 6 min’ walk distance.

**Conclusions:**

This study reports for the first time that specific VOC profiles can be found to differentiate IPF and CTD-ILD from both healthy controls and each other. Moreover, an ILD-specific VOC profile was strongly correlated with functional parameters. Future research applying larger cohorts of patients suffering from a larger variety of ILDs should confirm the potential use of breathomics to facilitate fast, non-invasive and proper differential diagnosis of specific ILDs in the future as first step towards personalized medicine for these complex diseases.

## Background

Fibrotic Interstitial lung diseases (ILDs) are a heterogeneous group of chronic lung diseases characterized by fibrotic remodeling of alveolar regions of the lungs. ILD comprise idiopathic ILD, of which idiopathic pulmonary fibrosis (IPF) is the most severe form [1], ILD of known cause such as drug-induced ILD or ILD associated with connective tissue disease (CTD-ILD), granulomatous ILD such as sarcoidosis, and other rare forms of ILD [2]. In the Paris area, the overall incidence of ILD is 19.4/100 000, while the most frequent diagnoses are sarcoidosis (42.6%), CTD-ILD (16%), and IPF (11.6%) [3]. Due to their relative low incidence and prevalence, ILDs are an under recognized problem within health care systems although these diseases are associated with considerable morbidity and mortality. If classified as a malignancy, IPF would rank as the eighth most prevalent cancer worldwide [1], while ILD are the leading cause of mortality in patients with CTD [4, 5].

Although unspecific pathways such as TGF-beta signaling, mechanotransduction and myofibroblastic differentiation of lung mesenchymal cells likely contribute to the terminal fibrotic process in all ILD [1], disease-specific injurious processes play key roles in the initiation of the fibrotic response [6–8]. In particular, while chronic pauci-inflammatory injury of the alveolar epithelium is believed to initiate IPF [9], activation of both the innate or adaptive immune systems and the subsequent inflammatory response are understood to play key roles in CTD-ILD [10].

Consistent with the distinct underlying pathogenic processes, response to therapeutics vary among ILDs. Most notably, while corticosteroids and immunosuppressant drugs are the cornerstones of treatment for immune/inflammatory ILD such as CTD-ILD [11], these drugs increase mortality and must be avoided in IPF [12] apart from acute exacerbations. By contrast, the anti-fibrotic drugs pirfenidone and nintedanib slow down functional decline and may increase survival in progressive fibrotic ILDs [1, 13–17] but to what extent they can also modulate inflammatory processes has only been limitedly explored until now [18, 19]. Consequently, in patients suffering from ILDs associated with high levels of inflammation co-treatment with immunomodulatory therapies besides these anti-fibrotic drugs might be beneficial [18]. Since such specific therapeutic requirements and responses are related to distinct ILDs, it is of crucial importance to obtain a differential diagnosis at the earliest possible moment to steer the appropriate medication per subgroup. In particular, discriminating IPF from immune/inflammatory ILD such as CTD-ILD is a common clinical conundrum. The difficulty of distinguishing CTD-ILD from idiopathic ILDs such as IPF can be exemplified by the facts that up to 15% of ILD patients also present symptoms compatible with CTD during their initial evaluation, whereas up to 25% of ILDs occur in patients with undiagnosed CTD [20].

Differential diagnosis of ILD ideally relies on the combination of clinical, imaging, alveolar lavage and serological data in the setting of a multidisciplinary discussion [21]. However, this process remains difficult and often requires analysis of surgical lung biopsies [22]. Indeed, surgical lung biopsy is still the single most informative test in cases where both the clinical and HCRT features fail to provide an exclusive diagnosis [23]. However, lung biopsy is often not possible due to a number of contraindications including age, comorbidities and the severity of the disease. There is thus a need for the development of other diagnostic markers that are less or even non-invasive, safe and fast.

A novel and currently untested approach for the non-invasive differential diagnosis of ILD could be the analysis of exhaled air, known to contain a complex mixture of volatile organic compounds (VOCs) that might be applied as potential biomarkers for chronic lung diseases [24, 25]. To this extent, we have applied a sampling methodology for collecting concentrated samples of exhaled air against which a thermal desorption gas chromatography—time of flight—mass spectrometry (GC-*tof*–MS) analysis has been employed [26]. Additionally, data analysis tools have been developed to enable the pipeline analysis of the generated GC–MS sample outputs [27]. By extracting informative VOCs from the compiled database and implementing them into a classifier of which the performance was evaluated [28], we have already shown that a specific VOC profile in breath can discriminate healthy controls from patients suffering from a variety of lung diseases including chronic obstructive pulmonary disease, ventilated-associated pneumonia and asthma [29–31]. However, it would clinically be more relevant to apply exhaled VOCs in differentiating between chronic lung diseases that (partly) share pathogenesis and symptoms yet display diverse outcomes and therefore require different treatment options. Therefore, the primary aim of the present study is to identify exhaled VOC profiles characteristic for IPF and CTD-ILD, in comparison to patients without lung disease as well as to each other. Such a unique volatile profile for the different ILDs could represent a novel diagnostic tool for positive and differential diagnosis of these complex diseases. Moreover, the secondary aim of this study is to investigate whether these exhaled VOCs, specific for either IPF or CTD-ILD, could be associated with lung function impairment and thus correlate with disease severity.

## Materials and methods

### Study design

A monocentric prospective observational cohort study was performed to compare VOC profiles in subjects without chronic lung disease (Controls) and in subjects with either IPF or CTD-ILD. Study subjects were recruited amongst patients referred for lung (IPF and CTD-ILD subjects) or renal function (control subjects) testing to the Physiology Department of Bichat-Claude Bernard university hospital (Paris, France) between January 2014 and March 2015.

All participants were fully informed, both written and orally, about the aim and details of the study and have given their written informed consent. Prior to the inclusion, the protocol of this study was approved by the regional ethical review board (CPP Ile de France IV, 2013-A01120-45). To answer the research questions, the present study consists of two groups of patients with either IPF or CTD-ILD as well as a group of control subjects without chronic lung diseases collected at the renal physiology department.

### Patients

#### Inclusion criteria

Subjects aged 40 to 80 and without chronic liver disease, HIV infection, diabetes, inflammatory bowel diseases or congestive heart failure were eligible for the study. Controls subjects were referred for the exploration of recurrent urinary lithiasis, had serum creatinine < 200 µM, had no known respiratory diseases including asthma earlier in life, no significant exertional dyspnea (Medical Research Council scale 0–1) and did not use any inhaled medications.

ILD was defined as the presence of reticulations, traction bronchiectasis, or ground-glass opacities on 2 high-resolution computed tomography (HRCT) scans performed at least 3 months apart. CTD was defined as either rheumatoid arthritis, Sjögren’s syndrome, polymyositis/dermatomyositis or undifferentiated CTD diagnosed according to American College of Rheumatology criteria [32, 33]. CTD-ILD was defined by the combination of ILD and CTD. IPF was diagnosed according to 2018 ERS/ATS/JAS/ALAT criteria [34]. IPF diagnoses were ascertained by reviewing medical charts established by the Referral Center for Rare Lung Diseases (Service de Pneumologie A, Paris, France) including information obtained after inclusion. All diagnoses were adjudicated by multidisciplinary discussion. Clinical and lung function data were obtained for patients in the two ILD groups according standard clinical protocols.

#### Clinical data

Lung function and HRCT data were collected from ILD patients. Vital capacity (VC), forced expiratory volume in 1 s (FEV1), total lung capacity (TLC) and carbon monoxide diffusion capacity (TLCO) using the single breath method were obtained according to ATS/ERS guidelines [35] and expressed relative to ECCS1993 reference values [36]. The 6-min walk test was performed according to American Thoracic Society guidelines [37] and the result was expressed as a fraction of the predicted value according to Enright [38]. HRCT scans were reviewed and classified as showing either one the patterns described in the 2018 ATS/ERS/JRS/ALAT IPF diagnosis statement (usual interstitial pneumonia-UIP, probable UIP, indeterminate for UIP or non UIP pattern) [34] or as the non specific interstitial pneumonia (NSIP) pattern, with or without subpleural consolidation, in the presence of predominant ground-glass opacities and lack of honeycomb lesions. Pathological analysis of lung biopsies or explanted lungs was obtained from medical records, when available.

##### Sampling and measurement of exhaled breath

For VOCs sampling, participants were asked to sit down, and to exhale tidally into a sterile 3L Tedlar bag (SKC Inc, Pennsylvania, USA) until the bag was full. The VOCs in the bag were transferred to a stainless steel two-bed desorption tube filled with carbograph 1TD/Carbopack X (Markes International, Llantrisant, Wales, UK). The desorption tubes were kept at room temperature until analysis. The desorption tubes were placed inside a TD100 automated thermal desorber for industry standard (Markes International, Llantrisant, Wales, UK) and heated to 350 °C to release the VOCs. Subsequently, 25% of the released VOCs were trapped at a cold trap at 5 °C, whereas 75% was re-stored at an identical stainless steel two-bed desorption tube for potential repeated measurement. Next, the released VOCs were injected in the GC-column at a temperature of 300 °C and separated by capillary gas chromatography (column: RTX-5 ms, 30 m × 0.25 mm 5% diphenyl, 95% dimethylsiloxane, film thickness 1 µm; Trace 1300GC, Thermo Fisher Scientific, Waltham, Massachusetts). The temperature of the gas chromatograph was programmed in the following manner: 40 °C during 5 min, then raised with 10 °C/min to a maximum temperature of 270 °C, which was maintained for 5 min. Time-of-flight mass spectrometry (*tof*–MS; Bench TOF-dx, Almsco International, Llantrisant, Wales, UK) was used to detect and identify compounds available in the samples. Electron ionization mode was set at 70 eV and the mass range *m/z* 35–350 was measured. Sample frequency of the mass spectrometer was set to 5 scans/sec and analysis run time to 33 min. Following this procedure, a chromatogram was generated for each subject.

### Data processing

After measurement, each chromatogram was processed to diminish the influence of non-biological variation. Denoising, baseline correction, alignment, normalization and scaling of the data were consecutively used based on the method previously described by Smolinska et al. [27]. Briefly, log transformation of the data was performed to convert heteroscedastic noise into homoscedastic noise [39] after which the chromatograms were denoised by a Daubechies wavelet with two levels of compression [40]. Baseline correction was done by B-splines with asymmetric least squares smoothing [41] and normalization of the chromatograms was carried out by probabilistic quotient normalization [42]. Peak picking was performed after which the area under a peak was calculated. Peaks for the same compound were identified and combined using correlation of the mass spectra. This resulted in a data matrix with individual participants as rows and individual peaks as columns.

### Data analysis

#### Selection of discriminatory VOCs

**Fig. 1:**
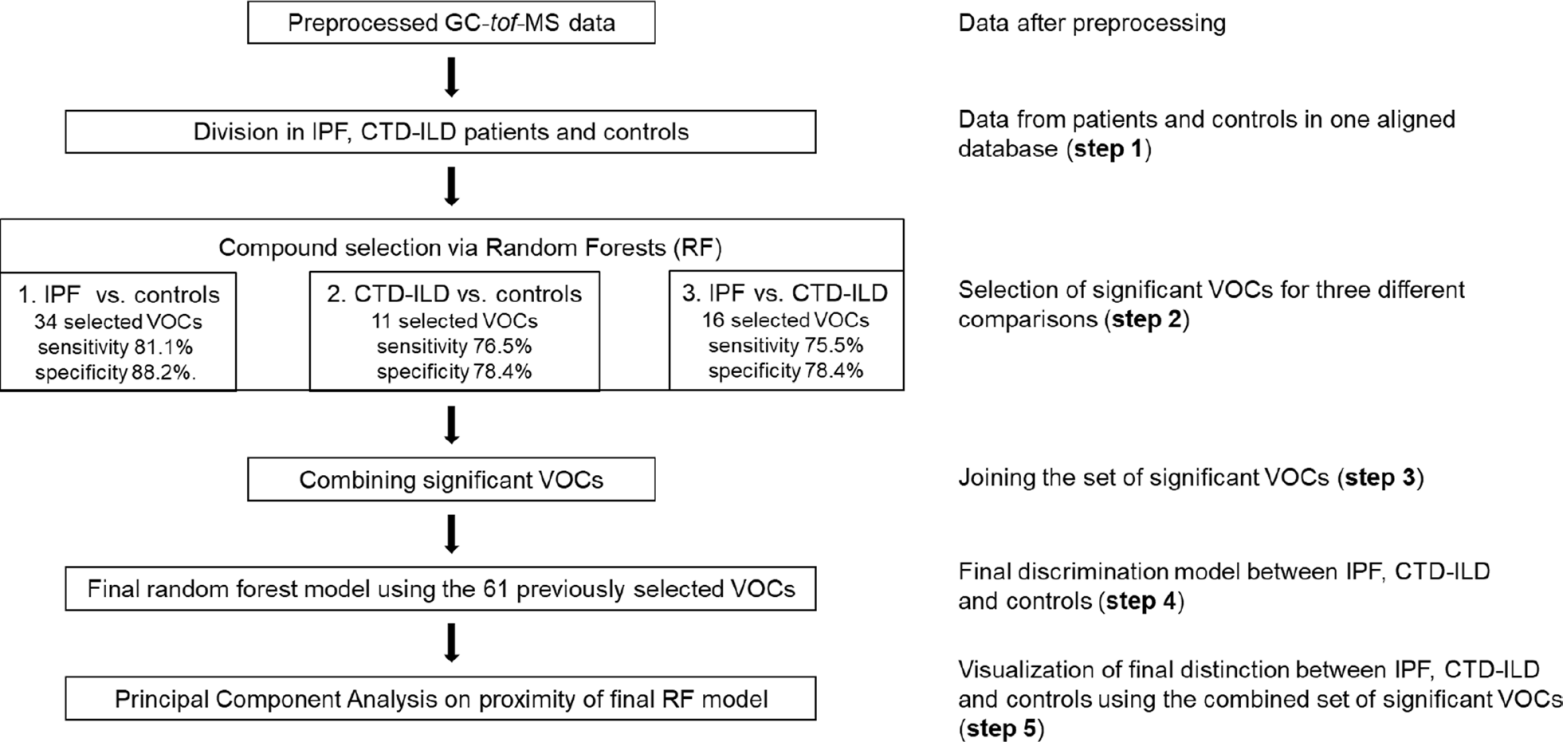
Conceptual flowchart presenting the approach used for statistical analysis. In **step 1**, a database is build with all clinical data and the preprocessed VOCs data contain three main groups: IPF (n =53), CTD-ILD (n=51) and healthy controls (n=51). In **step 2**, the machine learning method Random Forests (RF) was used to find discriminatory VOCs. For that purpose three different discriminatory RF models were built. Each discriminatory RF model was constructed on a training set (containing 80% of samples of each group) and validated using an independent test set (containing 20% of samples of each group). Training and test sets were selected using Duplex method (27). First RF algorithm was applied on VOCs data containing IPF and controls to find compounds linked to IPF. The second classification model was constructed on chromatograms belonging to CTD-ILD and healthy controls to allow selecting of VOCs related solely to CTD-ILD. The third RF algorithm was applied on data encompassing breath samples of IPF and CTD-ILD with the purpose to find VOCs differentially profiled between these two pulmonary pathologies. To demonstrate the performance of each RF analysis the receiver operating characteristic curve (ROC) is used and sensitivities and specificities determined. In **step 3**, the compounds selected as significant in step 2 are combined. In **step** **4**, the final RF model is constructed using chromatograms belonging to IPF, CTD-ILD and heathy controls. In order to demonstrate the differences between the three groups Principal Component Analysis (PCA) is performed on proximities obtained from the final RF model (**step 5**) with the purpose to visualize the relation between all breath samples

In order to apply VOCs for the identification of IPF and CTD-ILD patients, multivariate statistical modelling has been performed for selection of discriminatory VOC profiles unique for the various groups (see Fig. 1 for a conceptual flow chart of the data analysis). More specifically, three comparisons were performed. First, IPF patients were compared to a group of controls sampled at the renal department. Second, CTD-ILD patients were compared to the group of controls from the renal department. Finally, IPF and CTD-ILD patients were compared.

The selected multivariate statistical model was Random Forests (RF) [43], a machine learning algorithm that generates a large quantity of uncorrelated decision tree predictors to classify samples into the appropriate class. RF combines these decision trees to produce a generalization error called the out-of-bag error. This error demonstrates the accuracy of the model (accuracy = 1 – error) and is thus used to internally validate the distinct VOC profiles selected using RF. This error is always calculated using the samples that were never included in model optimization and development. Additionally, the model provides a measure of the importance of a variable that gives the most important variables the highest value. Based on this value, a subset of variables is chosen that can discriminate between two classes, in this case patients and controls. It is important to state that each discriminatory RF model was first constructed and optimised on a training set (containing 80% of samples of each group) and the final, optimized model containing only discriminatory VOCs was validated using an internal independent test set (containing 20% of samples of each group). Training and test sets were selected using Duplex method [27].

To demonstrate the efficacy of the binary classification RF model, a receiver operating characteristic (ROC) curve was created by calculating sensitivity and specificity using different thresholds for classifying positive class (i.e. CTD-ILD and IPF). The binary model was visualized using a score plot based on a principal component analysis (PCA) performed on the RF proximities. The proximities represent the similarity between individual samples as a result of the selected VOC profile: a small proximity value indicates similarity and a large proximity value dissimilarity between individual samples and thus between VOC profiles.

RF can also be directly used to simultaneously discriminate more than three classes. However, such multi-class models are more challenging to optimize, more difficult to interpret and often deliver larger classification errors than several binary RF models. Therefore, in order to represent the differences between all studied classes, a combination of binary classifiers was performed using hierarchical model fusion. Shortly, this approach creates a new set of scores for each sample by applying each of the binary classification model to the whole data set. In this study, the new scores are created by projecting the samples into the PCA score plot performed on the proximity matrix obtained from each of the binary model (i.e. IPF vs. control, CTD-ILD vs. control and IPF vs. CTD-ILD). Those new sets of scores can then be combined as new coordinates and will visualize the differences between groups. This described strategy is only valid if each of the binary classification models is first properly optimized and validated.

#### Chemical identification of VOCs

**Fig. 2:**
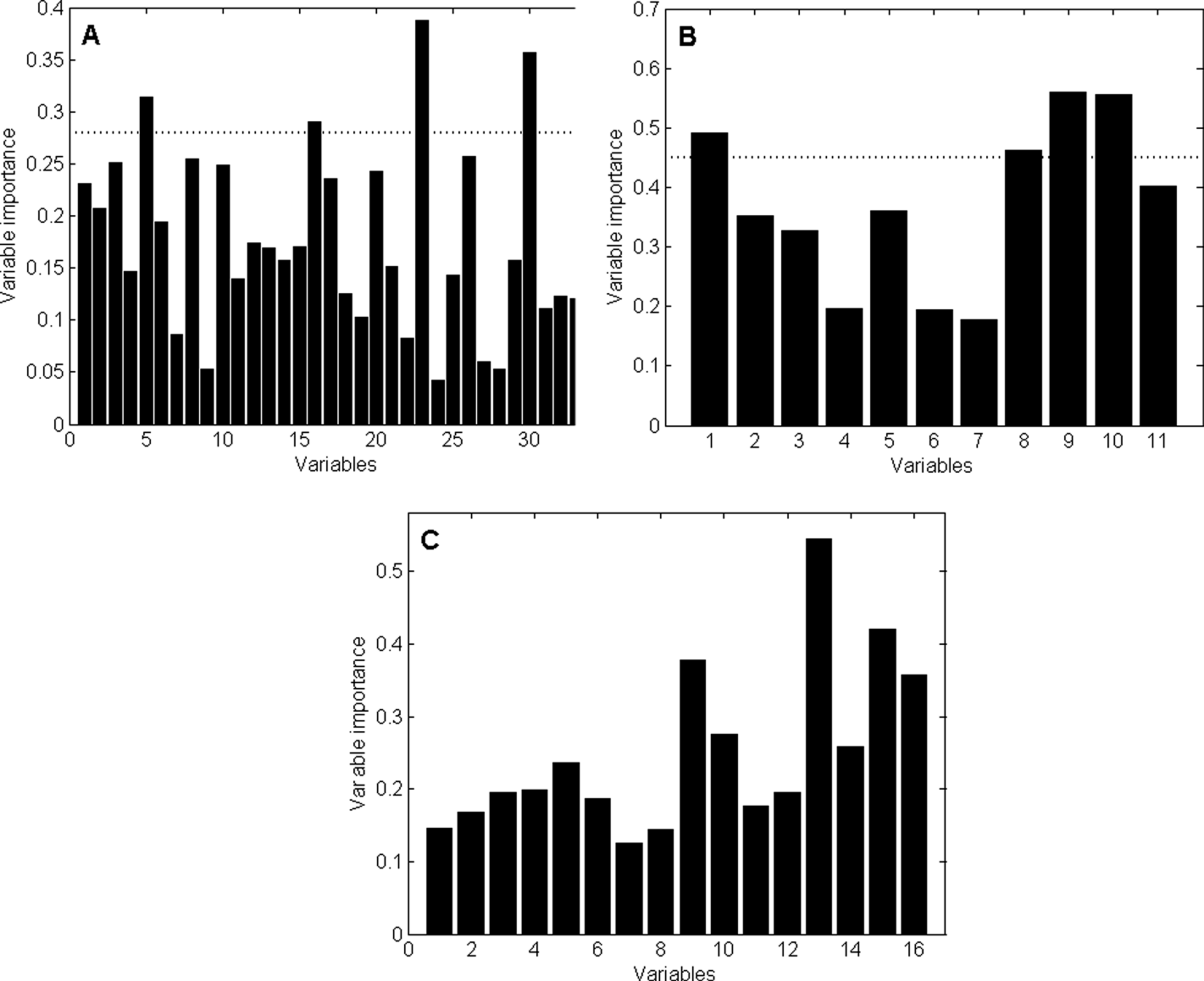
The importance of the variables for each of the three comparisons. The dashed horizontal lines indicate the chosen cut-off to select the most important VOCs for chemical identification. **A** IPF vs. controls; **B** CTD vs. controls; **C** IPF vs. CTD-ILD

For the two comparisons between the individual diseases and the controls, the most important VOCs were selected for chemical identification. The measure of importance for each individual VOC in the two discriminatory profiles was determined using the RF-variable importance, where the VOCs with the highest value had the largest contribution to the model. The selection of VOCs for chemical identification was based on defining a cut-off point to separate the VOCs clearly standing out from the rest of the volatiles in the model. The plots displaying the RF-variable importance for the discriminatory VOCs in both models as well as the selected cut-offs are displayed in Fig. 2A, B.

Additionally, all VOCs of the comparison between IPF patients and CTD-ILD patients were chemically identified due to their clinical relevance (see Fig. 2C for the relative contribution of these VOCs to the discriminatory profile). The identities of these VOCs were determined in two ways: (1) spectrum recognition using the National Institute of Standard and Technology (NIST) library in combination with an in-house composed compound database in which pure compounds were previously recorded and (2) validation of the identification described in step 1 by an experienced mass spectrometrist as described earlier [30].

#### Influence of confounders

Since differences in age and gender can influence the classification between groups [44], it is important to rule out that these factors influenced the classification model in this study. The factors that were found significant between the different groups (i.e. age, gender and smoking status) were tested by regularized MANOVA [45] to determine whether they influenced the models significantly. A p value < 0.05 was considered statistically significant.

#### Correlation between VOCs and lung function parameters

Canonical correlation analysis (CCA) [46], which can be considered an extension of the binary Pearson correlation analysis, was used to calculate a correlation between a set of compounds (in this case the whole set of discriminatory VOCs) and characteristics (in this case the corresponding lung function parameters of the same patients). A p-value of < 0.05 was considered statistically significant. These lung function parameters include VC, TLC, FRC, FEV1, DLCO, PaO_2_, PaCO_2_ and 6MWD. Before CCA analysis, both datasets were log-transformed. A subset selection of the lung function parameters was made based on the contribution of the VOCs to the CCA model to achieve the best possible correlation. A correlation coefficient and corresponding p-value were reported in combination with figures of the correlation.

## Results

### Patients

The study included 155 subjects of whom 104 were patients and 51 controls. The control subjects were referred for recurrent urinary lithiasis. Fifty-three patients with IPF and 51 patients with CTD-ILD were included. The characteristics of study subjects are summarized in Table 1.

**Table 1:** Characteristics of all subjects analyzed in the present study

	**Controls**	IPF patients	CTD-ILD patients	Significance Ctrls vs. IPF	Significance Ctrls vs. CTD-ILD	Significance IPF vs. CTD-ILD
Number of subjects	51	53	51	ns	ns	Ns
Age (yrs. ± STD)	53 (± 10)	69 (± 9)	57 (± 12)	5.8*10^–6^	ns	1*10^–6^
% male	68	75	26	ns	0.002	1.63*10^–6^
% smokers ex/current	35/16	60/0	24/4			0.02
mMRC class 0	44	5	5			ns
mMRC class 1	7	29	22			ns
mMRC class 2	0	12	16			ns
mMRC class 3	0	6	5			ns
mMRC class 4	0	1	3			ns
VC (% ± STD)	NA	77 (± 22)	78 (± 23)			ns
TLC (% ± STD)	NA	71 (± 15)	76 (± 15)			ns
FRC (% ± STD)	NA	74 (± 17)	78 (± 15)	ns	ns	0.001
FEV1 (% ± STD)	NA	80 (± 22)	78 (± 25)	ns	ns	0.0008
DLCO (% ± STD)	NA	49 (± 17)	49 (± 17)	ns	ns	0.03
PaO2 (± STD mm Hg)	NA	75 (± 9)	80 (± 12)			ns
PaC02 (± STD mm Hg)	NA	41 (± 4)	39 (± 4)			ns
6MWD (% ± STD)	NA	88 (± 20)	78 (± 20)			ns

Continuous variables were displayed as mean and standard deviation or percentages. Lung function parameters are displayed as a percentage of the predicted value based on age and gender. *mMRC* modified medical research council dyspnea scale *VC* vital capacity, *TLC* total lung capacity, *FRC* functional residual capacity, *FEV1* forced expiratory volume in 1 s; *DLCO* diffusing capacity for carbon monoxide, *PaO*_*2*_* and PaCO*_*2*_ oxygen and carbon dioxide pressure in arterial blood, *6MWD* six-minute walk distance, *NA* not available. Significances were calculated using a Student’s t-test (the data are normally distributed based on Lilliefors test) in combination with False Discovery Rate correction. *ns* not significant

All patients in the CTD-ILD group suffered from fibrotic ILD. In this group, HRCT showed a NSIP pattern in 26 patients (including 4 with areas of consolidation and 2 with mosaic attenuation), an indeterminate for UIP pattern in 9 patients, a probable UIP pattern in 7 and an UIP pattern in 4 patients. Criteria-defined underlying CTD was rheumatoid arthritis in 11 patients, dermatomyositis in 12 patients, undifferentiated CTD in 11 patients, and primary Sjogren’s syndrome in 8 patients.

In the IPF group, HRCT showed an UIP pattern in 30 patients, a probable UIP pattern in 17 patients, and an indeterminate pattern in 4 patients. Pathological analysis of lung tissue was available in 7 patients, and showed an UIP pattern in 5 patients, a probable UIP pattern in 1 patient, and an indeterminate pattern in 1 patient. Among the 23 patients without a UIP HRCT pattern, a final diagnosis of IPF was ascertained by surgical lung biopsy in one patient showing a probable UIP histopathological pattern, retrospectively by pathological analysis of explanted lungs in 4 patients, and by MDD in the others. Three patients had “likely IPF” according to the 2018 IPF diagnosis criteria [34].

For each of the study parameters, a student’s t-test was performed to check for significant differences between groups. Multiple testing correction was performed using the False Discovery Rate correction [47].

The difference in age of the participants between the controls and IPF patients (p-value: 5.8^−06^) and between the IPF and CTD-ILD patients (p-value: 1.0^−06^) was significant. Additionally, the gender distribution between the controls and CTD-ILD patients (p-value: 0.002) and the IPF and CTD-ILD patients (p-value: 1.63^−06^) was significantly different. Finally, the distribution of current and ex-smokers was significantly different between the IPF and CTD-ILD patients (p-value: 0.02). Among the 51 CTD-ILD patients, 40 were treated with oral corticosteroids, 25 received cytotoxic immunosuppressants (either cyclophosphamide, azathioprine, methotrexate, or mycophenolate) while 16 were treated with proton pomp inhibitors (PPI). Among the 53 IPF patients, 30 were treated with pirfenidone, 6 were treated with nintedanib, 6 were treated with corticosteroids, 12 received PPI, and none received cytotoxic immunosuppressants. Among the 51 controls, 14 received no medication whereas 8 were treated for hypertension; the most common drug classes were centrally active antihypertensive drugs (4 patients) and calcium channel blockers (3 patients).

### VOC profiling for IPF patients versus controls

**Fig. 3:**
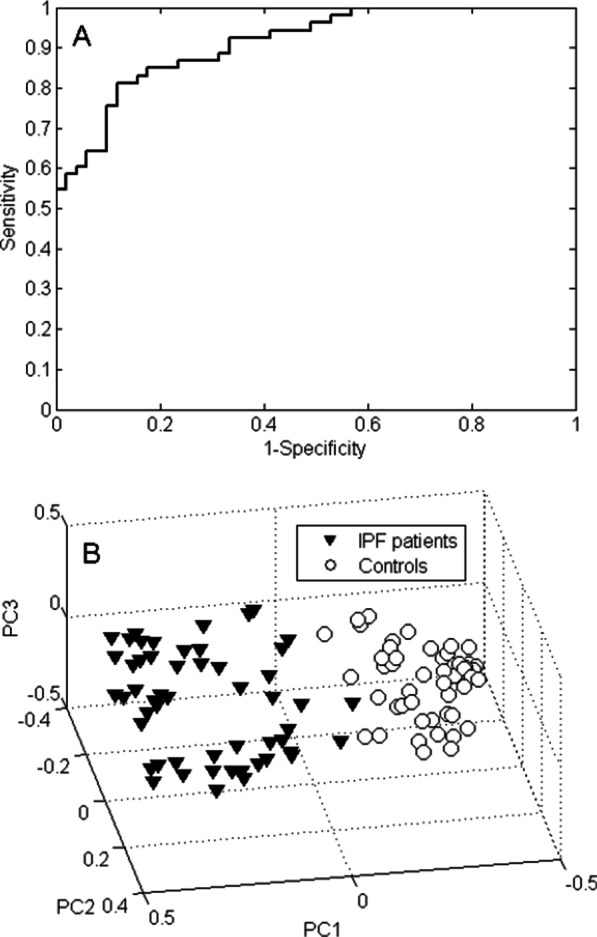
VOC profiling for IPF versus controls. **A** ROC curve of the 34-VOC IPF versus controls profile. The AUC is 91.2%. **B** 3D PCA plot of Random Forests proximities comparing IPF and controls. The distance between individual points expresses their similarity, i.e. short distance indicates s highly similar VOC profile and vice versa

The exhaled VOCs from the 53 IPF patients were compared with those present in the breath of the 51 controls. A total of 34 VOCs was selected that could discriminate the IPF patients from the controls. The IPF versus control profile had 84.6% accuracy, sensitivity of 81.1% and specificity of 88.2%. The Receiver Operating Characteristic (ROC) curve of the IPF versus controls using the 34 discriminating VOCs had an area under the curve (AUC) of 91.2% (Fig. 3A). A PCA score plot was generated to display groupings in the data as a result of the selected subset of VOCs. This score plot is depicted in Fig. 3B and shows clear separation between both groups. The chemical identity of the 5 most contributing VOCs is given in Table 2, which shows that the concentration of all but benzaldehyde is lower in the IPF patients.

**Table 2:** Chemical putative identities of the most contributing VOCs of the comparison between IPF and controls

Chemical identity	CAS number	Change in IPF with respect to controls
Ethanol	64-17-5	Down
Heptane	142-82-5	Down
Benzaldehyde	100-52-7	Up
Unknown	NA	Down
Dimethyl sulfide	75-18-3	Down

### VOC profiling for CTD-ILD patients versus controls

**Fig. 4:**
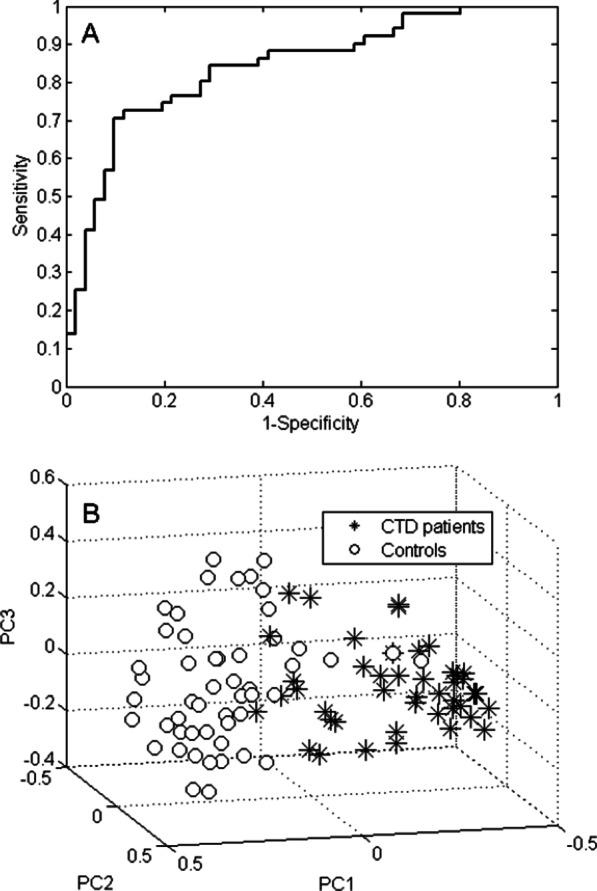
VOC profiling for CTD-ILD patients versus controls. **A** ROC curve of the 11-VOC CTD-ILD versus controls profile. AUC is 83.9%. **B** 3D PCA plot of Random Forests proximities comparing CTD-ILD patients and controls

When comparing the exhaled VOCs of the 51 included CTD patients with those present in the breath of the controls, 11 VOCs were selected as discriminatory. The 4 most contributing VOCs in this model are listed in Table 3, all of which decreased in concentration in the CTD-ILD patients compared to the controls. This discriminatory VOC profile provided a classification accuracy of 77.5% with a sensitivity of 76.5% and a specificity of 78.4%. The corresponding ROC curve and PCA scores plot are displayed in Fig. 4A, B. The ROC AUC was 83.9%.

**Table 3:** Chemical putative identities of the most contributing VOCs of the comparison between CTD-ILD and controls

Chemical identity	CAS number	Change in CTD-ILD with respect to controls
2-Heptanone	110-43-0	Down
4-penten-ol	821-09-0	Down
2,5-dimethyl furan	625-86-5	Down
Ethanol	64-17-5	Down

### VOC profiling for IPF patients versus CTD-ILD patients

**Fig. 5:**
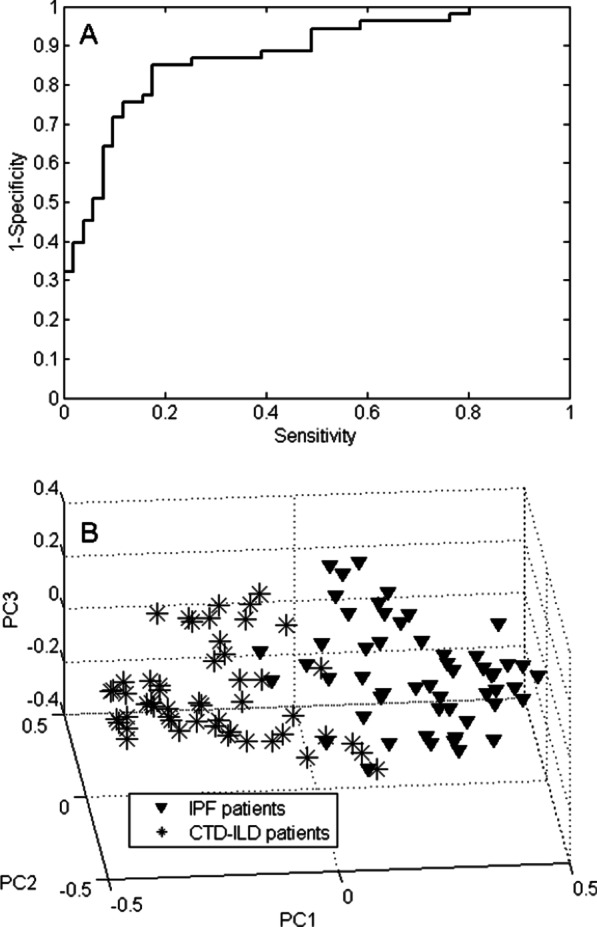
VOC profiling for IPF patients versus CTD-ILD patients. **A** ROC curve of the 16-VOC IPF versus CTD-ILD profile. AUC is 83.8%. **B** 3D PCA plot of Random Forests proximities comparing IPF and CTD-ILD patients

A subset of 16 VOCs was able to discriminate IPF from CTD-ILD with an accuracy of 76.9%, a sensitivity of 75.5% and a specificity of 78.4%. The ROC curve (Fig. 5A) had an AUC of 83.8% and the PCA scores plot (Fig. 5B) displays a separation between the two patient groups. The chemical identity of all 16 VOCs is displayed in Table 4. Seven VOCs were exhaled in lower concentrations in the IPF patients compared to the CTD-ILD patients, whereas 9 VOCs were increasingly exhaled.

**Table 4:** Chemical putative identities of all discriminatory VOCs of the comparison between IPF and CTD-ILD

Chemical identity	CAS number	Change in IPF with respect to CTD-ILD
Acetone	67-64-1	Down
Dimethylsulfone	3877-15-4	Up
Heptane	142-82-5	Down
4-methyl-2-heptene	3404-56-6	Up
Branched C11H24	NA	Up
Undecane	1120-21-4	Down
Tridecane	629-50-5	Up
Octadecane	593-45-3	Down
Branched C12H24	NA	Up
Pyrrolidine	123-75-1	Down
Decanal	112-31-2	Down
2-heptanone	110-43-0	Up
Branched C14H30	NA	Up
4-penten-ol	821-09-0	Up
2,5-methylfuran	625-86-5	Down
2-thiapentane	3877-15-4	Up

### VOC profiling of controls, IPF and CTD-ILD patients

**Fig. 6:**
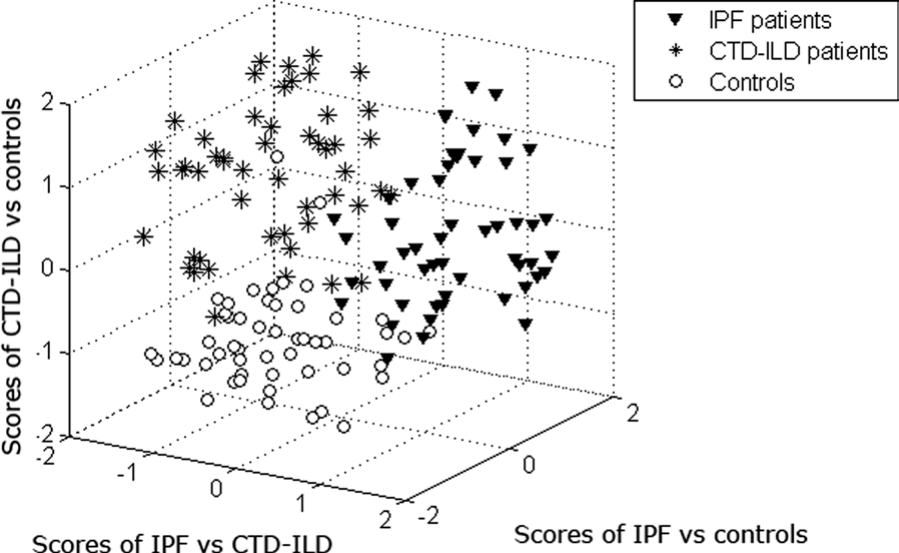
VOC profiling of IPF versus CTD-ILD versus controls. 3D score plot of combined binary classification RF model

In order to visualize the outcomes of all three RF models, a set of new scores was created using hierarchical model fusion as previously described by Smolinska et al. [48]. Those new scores (each coming from one of the binary classification model) were applied to create a score plot as shown in Fig. 6. The discriminatory VOC profiles underlying this combined model were not influenced by the differences in age, gender, or tobacco history (Table 5).

**Table 5:** The influence of the significant study parameters on the discriminatory VOC profiles

Study Parameter	Comparison	p-value
Age	Controls vs. IPF	0.212
Age	IPF vs. CTD-ILD	0.219
Gender	CTD-ILD vs. Controls	0.072
Gender	IPF vs. CTD-ILD	0.814
Smoking	IPF vs. CTD	0.637

Regularized MANOVA was used to test whether significant study parameters were influential. A p-value < 0.05 was considered significant

### Correlation between discriminatory VOCs and disease severity

**Fig. 7:**
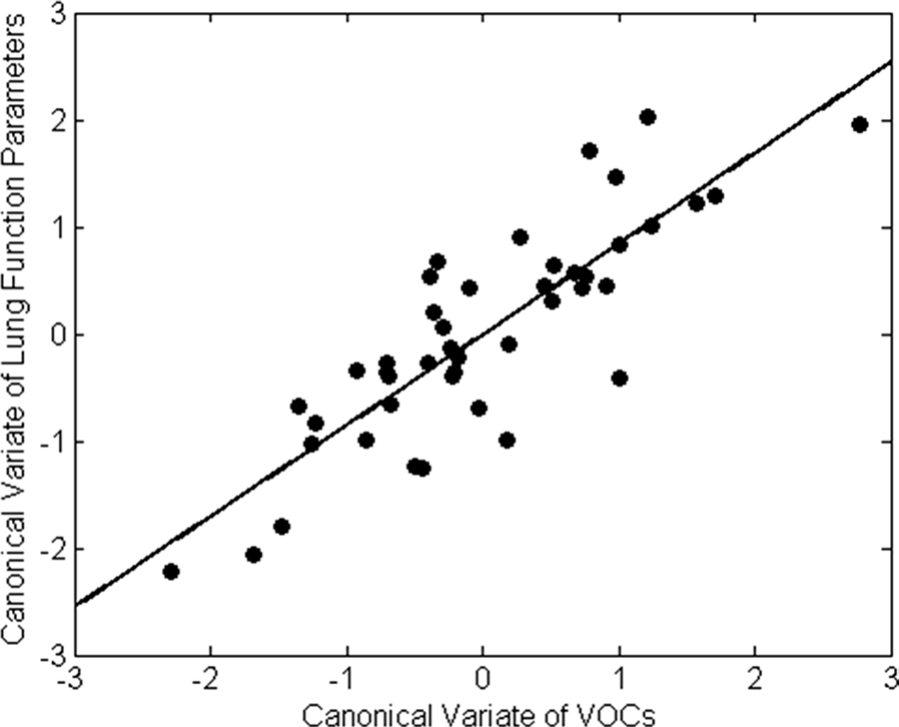
Correlation between the discriminatory VOCs and lung function parameters TLC and 6MWD. This correlation plot depicts the canonical variate of the VOCs on the x-axis and the canonical variate of the TLC and 6MWD on the y-axis

To examine the clinical relevance of the identified VOCs, correlation between the discriminatory volatiles of both groups of patients and the lung function parameters characteristic for IPF and CTD-ILD was examined using CCA. As depicted in Fig. 7, a significant correlation was observed between the previously selected VOCs and two of the included functional parameters, i.e. TLC and 6MWD. A correlation coefficient of 0.8484 with a corresponding p-value of 0.0308 was achieved, indicating that functional impairment is positively associated with the selected discriminatory VOCs and thus with the observed ILD volatile profiles.

**Fig. 8:**
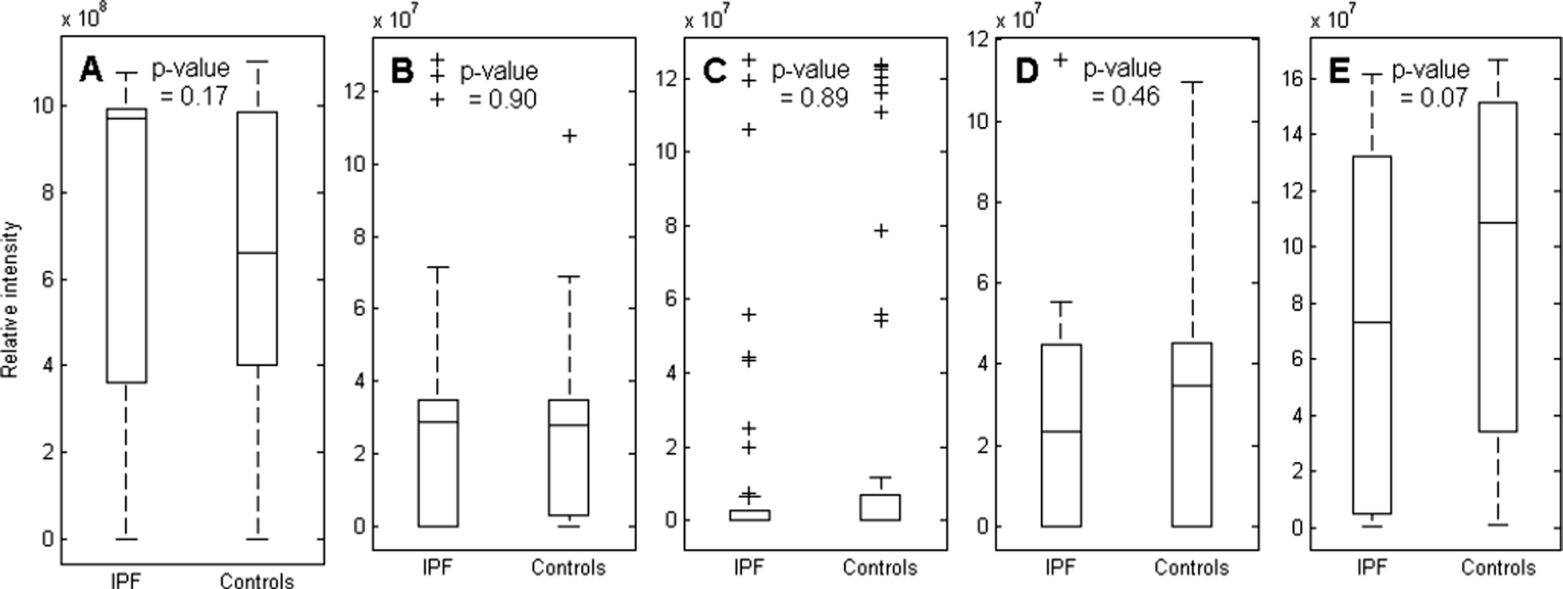
Relative concentrations of individual VOCs reported in literature to differ in the breath of IPF patients and healthy controls. The displayed boxplots represent the following volatiles: **A** Isoprene, **B** p-Cymene, **C** Ethylbenzene, **D** m- and/or p-Xylene, **E** o-Xylene. In each plot, the p-value is displayed, where a p-value < 0.05 is considered significant. m-, p-, and o-xylene are hard to distinguish from ethylbenzene, leading to possible misidentification, thus their significances are also reported

## Discussion

Rapid, accurate and ideally non-invasive diagnosis of ILD is a key challenge in respiratory medicine. Until now, invasive lung biopsies are often still required for the correct differential diagnosis in ILDs as other currently available diagnostic tools including imaging techniques (e.g. HRCT) and biological markers (e.g. chemokines, proteases and growth factors, [49] fail to be exclusive [22, 23]. Therefore, the possible usefulness of non-invasive volatile markers excreted in the breath to identify specific types of ILD has been explored in the present study.

We report an attempt to find discriminatory VOC profiles in the breath of patients suffering from IPF or CTD-ILD and subjects without chronic lung disease. In the present study, 34 VOCs correctly discriminated IPF patients from healthy controls with 84.6% accuracy, whereas 11 VOCs discriminated CTD-ILD patients versus healthy controls with 77.5% accuracy. Moreover, the two ILDs were correctly distinguished from each other with an accuracy of 76.9% using a set of 16 VOCs. Interestingly, this last subset of 16 volatiles was strongly correlated with two clinical parameters of the diseases, i.e. total lung capacity and 6 min’ walk distance, indicating the possible pathological relevance of the selected VOCs in ILDs.

Chemical identification of the most discriminatory VOCs observed in this pilot study leads to interesting observations. For instance, 5 volatiles have important discriminative power in more than one of the classification models. Both 2-heptanone and 4-pentan-1-ol are found in increased levels in IPF compared to CTD-ILD and display decreased relative concentrations in CTD-ILD versus healthy controls, indicating that these two VOCs might be related to CTD-ILD. Similarly, the relative concentrations of heptane are decreased in the breath of IPF patients compared to both the healthy controls and CTD-ILD patients, suggesting that this volatile is probably related to IPF pathogenesis. The remaining two VOCs that were of interest in more than one of the comparisons are dimethylsulfide, dimethylsulfone and 2,5- dimethylfuran. Dimethylsulfide is decreased in IPF compared to controls whereas the levels of dimethylsulfone, which is formed upon oxidation of dimethylsulfide by hydrogen peroxide, are increased in IPF compared to CTD-ILD. The presence of these two volatiles can be explained by enhanced production of hydrogen peroxide by NADPH oxidase 4, activated by transforming growth factor β which is considered a hallmark of IPF [50]. Finally, 2,5 dimethyl furan is a biomarker of smoking [26] and involved in singlet oxygen scavenging [51]. The fact that the levels of this volatile are decreased in CTD-ILD compared to controls as well as in IPF versus CTD-ILD indicates that these lower levels are not merely a reflection of the different inclusion rates of current and ex-smokers in all patients groups, but also associated with the oxidative stress underlying the pathology of ILD, and IPF in particular.

Within the IPF-specific profile, benzaldehyde levels were increased whereas the levels of ethanol, heptane and dimethylsulfide were decreased in comparison to controls. Benzaldehyde is a naturally occurring dietary chemical, present in for instance almonds, which is also used as food additive and in scented products and cosmetics [52, 53]. Endogenously, benzaldehyde is formed out of benzylamine, a metabolite of monoamine oxidase inhibiting drugs, by semicarbazide-sensitive amine oxidase [54] which is a pro-inflammatory enzyme particularly expressed in the lungs [55] and elevated in smokers and patients suffering from inflammatory diseases [56]. Although there are no reports yet on the specific role of benzaldehyde in IPF, it has been shown in animal models that inhibition of amine oxidase reduces pulmonary inflammation and the development of fibrosis [56, 57]. Therefore, amine oxidase might be involved in IPF, causing the observed elevated levels of benzaldehyde in the exhaled breath of IPF patients. Alternatively, exogenous benzaldehyde is absorbed through skin as well as via the lungs. Upon being metabolized by aldehyde dehydrogenases to benzoic acid, conjugates will be formed with glycine or glucuronic acid and excreted in the urine [58]. Higher benzaldehyde levels could therefore also reflect an impaired liver metabolism in IPF patients, leading to higher levels of this metabolite in the circulation and thus breath.

The relative concentrations of heptane were reduced in the breath of IPF patients. Since heptane is a known marker of oxidative stress [59, 60] and reported to be increased in patients suffering from various lung diseases, including tuberculosis and lung cancer [61, 62], the observed decrease in IPF, a disease associated with oxidative stress as well, is remarkable and difficult to interpret. Finally, the relatively lower levels of dimethyl sulfide in the breath of IPF patients can be explained by the recent finding that this volatile offers protection against oxidative stress and ageing, two processes associated with IPF pathology, by serving as a substrate for the antioxidative enzyme called methionine sulfoxide reductase A [63].

In the breath of CTD-ILD patients, a decrease was noticed in the relative concentrations of heptanone, 4-penten-ol and 2,5-dimethylfuran compared to healthy controls. A clear explanation for the lowered levels of heptanone and 4-penten-ol is currently still lacking, although an isomer of the latter (i.e. 4-penten-2-ol) has already been reported as a marker for lung cancer [61].

The only volatile displaying the same pattern in both ILDs was ethanol, whose relative concentration was decreased in both IPF and CTD-ILD compared to healthy controls. In the human body, ethanol is constantly formed as a metabolite of acetaldehyde which is in situ generated during the metabolism of pyruvate, threonine, deoxyribose-5-phosphate and other substrates [64]. Interestingly, this endogenous formation of ethanol is under influence of various physiological circumstances and can be hampered by both ageing and oxidative stress [64], two conditions frequently reported to be associated with ILDs in general and IPF specifically [8, 65]. Second possible source of pulmonary ethanol secretion is the lung microbiome as a study of Bos et al*.* has revealed that bacterial DNA fragments can be linked to enzymes implicated in the production of VOCs predictive of respiratory tract colonization and/or infection including ethanol [66]. Interestingly, the recent work of Gupta et al. suggests that each respiratory disease not only has a specific disease etiology but is also associated with unique microbial signatures [67]. Within ILD, a higher relative abundance of Streptococcus and Staphylococcus was observed as has also previously been reported to contribute to disease progression in IPF [68]. Additionally, a significantly higher abundance of Haemophilus, Stenotrophomonas and Enterobacteriaceae was shown by Gupta et al.[67]. Whether such ILD-specific alterations in the pulmonary micobiome were indeed involved in the affected ethanol excretion in our patients remains to be investigated, but our observation that exhaled ethanol levels were not discriminatory between the two investigated ILDs certainly fits within this hypothesis.

Most exhaled studies regarding ILDs have focused on either the fraction of exhaled nitric oxide (FE_NO_) or markers in exhaled breath condensate (EBC). The few studies that have focused on identifying FE_NO_ in ILD display rather conflicting results [69–71], which might not be that surprising considering the fact that FENO is a marker of inflammation, a process that is not a mandatory contributor to ILD pathology [72]. Within the EBC studies performed over the last years, focus was mostly on measuring markers of oxidative stress including malondialdehyde [73], nitrite [74], 8-isoprostane [75] and hydrogen peroxide itself [75]. However, all these markers are rather general for the occurrence of oxidative stress, a process involved in the pathology of many chronic diseases, and thus never shown to be exclusively different for specific ILDs. Moreover, these markers have always been analyzed on individual level, which will obviously also hamper their usefulness to differentiate between ILDs as these multifactorial diseases cannot be characterized by a single marker or process. Recently, enhanced levels of various collagen-related amino acids including proline, 4‐hydroxyproline, alanine, valine, leucine and allysine have been detected in EBC, and confirmed to some extent in the exhaled breath, of IPF patients [76]. Interestingly, all significantly altered compounds were strongly correlated to each other yet independent from commonly used lung function parameters including FVC and DLCO. These findings suggest a shared metabolic process underlying the elevated amino acid levels that is related to ongoing or newly developing fibrotic processes rather than already present fibrotic tissue [76, 77]. Although this is an intriguing observation, it is still related to only one single process in which other important matrix metabolites in IPF are not yet included and has not yet proven exclusiveness for IPF compared to other ILDs or chronic lung diseases associated with bronchial fibrosis such as COPD and asthma [77].

Recently, Enose technology was shown to distinguish ILD patients from healthy controls and to discriminate between different ILD subgroups [78]. Although this is a very promising result that indicates breath analysis might be useful for timely diagnosis of specific ILDs in the future, such Enose studies i) do not provide insight into the biological mechanisms of diseases and ii) generate device-specific data that are hardly translatable to other devices or technologies. Consequently, there is still a need for discriminative breathomics to diagnose and monitor ILDs. Until now, only two of such comprehensive breathomic studies have examined the excretion of disease-specific VOCs in ILDs [79]. In the first study in 2005, elevated level of ethane were reported to be exhaled in the breath of 34 ILD patients compared to the exhaled air of 16 healthy controls [80]. Interestingly, the ethane levels were correlated with clinical outcome parameters as well as with lactate dehydrogenase, an indicator of oxidative stress [80]. The other study measured exhaled VOCs in 40 IPF patients and identified 5 VOCs (i.e. isoprene, ethylbenzene, p-cymene, acetoin and an unknown compound) that were significantly different in their breath compared to that of 55 healthy controls [81]. Isoprene, ethylbenzene and p-cymene were also detected in our study, as well as m-, p-, and o-xylene as they are difficult to distinguish from ethylbenzene. Their relative concentrations in our study and the corresponding p-values are depicted in Fig. 8. None of these individual VOCs displayed a significantly altered level in the breath of IPF patients compared to that of healthy controls, although o-xylene almost reached significance (p-value 0.067). These discrepancies could be explained by differences in methodology and an alleged small effect size due to the heterogeneity of the disease. Alternatively, these differences may arise from the fact that we employed an age-matched control group whereas Yamada et al. included a control group that was half the age of the patient group. Moreover, we have measured all excreted VOCs followed by selecting discriminating VOC profiles rather than measuring a subselection of volatiles of which the significant change is analyzed on an individual level. Nevertheless, the observation of Yamada et al*.* that changes in VOCs are related to clinical parameters including lung function underlines the usefulness of breathomics in diagnosing IPF [81]. Similarly, we also observed a significant correlation between lung function parameters and the discriminatory VOC profiles for both ILDs. As a compromised lung function is the key clinical feature of ILDs, this observed correlation indicates that the selected VOCs may be linked to the general pathogenesis of these diseases. Future research has to elucidate whether this link is specific per ILD or general for all diseases with impaired lung function (including ILDs) and whether it can be used to develop breath biomarkers of prognosis, disease severity and therapy efficacy. Developing biomarkers of disease progression and therapeutic efficacy is of special interest as monitoring of ILD patients currently relies mostly on clinical, morphological and functional criteria, which may lack sensitivity in detecting early or minimal changes in disease activity. It can be anticipated that developing biomarkers of disease progression and therapy efficacy will aid in phenotyping ILD patients, i.e. creating subgroups of patients based on their disease severity and response to therapy, as a first step towards precision medicine in ILD [82].

Although an effect of age, gender and tobacco history and main medication use on the discriminating volatiles was not observed in the present study, it is important to mention that it was impossible to check the complete influence of medication as most patients were on a mixture of different medications and the patient groups were too small to take every possible drugs regime into account. Since medication has previously been described as possible confounding factor influencing VOC content in human breath [44], future studies should include larger patient groups to exclude any effect of the different medication regimes commonly applied within ILD treatment. Indeed, the effect of either anti-inflammatory and immunosuppressive agents, or antifibrotic agents, may explain some of the difference in VOC profiles between CTD-ILD and IPF patients. Additionally, such future studies could also stratify subgroups on age, gender and tobacco history to explicitly search for specific discriminative VOCs correlated with these factors associated with the pathophysiology of the disease. By including larger cohorts of more subgroups of ILD in future studies it can also be investigated whether possible overlap in exhaled volatiles exists as only small differences in VOC profiles can be expected if the clinical separation between subgroups of patients is very small as well. Interestingly, despite the lack of further subgrouping due to the relatively low patient numbers in the present study, we did not observe any overlap between the various patient groups upon visual analysis of the data using untargeted PCA analysis. This promising observation indicates no patients had a VOC profile resembling the exhaled volatiles belonging to a diagnosis different from the one they were included for.

The control subjects included in the present study could not be considered healthy since they were referred for recurrent urinary lithiasis. Additionally, some of the control subjects might already suffered from undiagnosed lung diseases as also ex- and current smokers were included to account for tobacco smoking being an important risk factor or developing ILD. Since they did not report any pulmonary symptoms at the time of inclusion, it was anticipated they did not suffer from clinically significant lung damage as observed in and related to ILD and would thus be a suitable control group to find discriminative VOCs related to ILD pathology instead of smoking. Indeed, the possible presence of undiagnosed chronic lung diseases in the control group would actually strengthen the clinical use of the observed discriminative volatiles for IPF and CT-ILD as they are then definitely not related to general chronic lung damage or pathways involved herein including inflammation. To further optimize the specificity of these discriminative VOCs, future studies should include an age-matched control group with other acute and chronic underlying pulmonary conditions. The diagnosis of IPF relied on MDD in 16/51 patients, which occurred mostly in patients with a probable UIP HRCT pattern and without histopathological data. Although this is a limitation to the study, review of follow-up charts allowed to ascertain IPF in all patients because of either (1) histopathological data confirming IPF, (2) further imaging showing a complete UIP pattern or (3) the lack of any alternative diagnosis at follow-up. In the present study, 56% in the IPF group had a UIP HRCT pattern, which is similar to the Europe-wide IPF network registry (83).

Finally, although all classification models were internally validated using a test set, external validation should also be added to future studies to minimize the risk of overfitting the data and to maximize the certainty of the discriminative compounds and their universal power.

## Conclusion

In conclusion, this pilot study reports for the first time that VOC profiles can be detected in the breath of patients suffering from IPF or CTD-ILD that differentiate them from each other as well as from age-matched healthy controls. Moreover, an ILD-specific VOC profile was strongly correlated with clinical parameters. Future research applying larger cohorts of patients suffering from various types of ILDs and including external validation sets should confirm the potential use of breathomics to facilitate fast, non-invasive and proper differential diagnosis of specific ILDs in the future as first step towards personalized medicine for these complex diseases.

## Data Availability

The datasets used and/or analyzed during the current study are available from the corresponding author on reasonable request.

## Abbreviations

6MWD: 6 Minute walking distance
AUC: Area under the curve
CCA: Canonical correlation analysis
CTD-ILD: Connective tissue disease associated ILD
DLCO: Diffuse lung capacity for CO
EBC: Exhaled breath condensate
FE_NO_: Fraction exhaled NO
FEV_1_: Forced expired volume in 1 s
GS-*tof*–MS: Gas spectrometry time of flight mass spectrometry
HRCT: High resolution computer tomography
ILD: Interstitial lung disease
IPF: Idiopathic pulmonary fibrosis
NIST: National institute of standard and technology
PCA: Principal component analysis
RF: Random forest
ROC: Receiver operating characteristic
TGF: Transforming growth factor
TLC: Total lung capacity
UIP: Usual interstitial pneumonia
VOC: Volatile organic compound

## References

[1] Antoniou KM, Margaritopoulos GA, Tomassetti S, Bonella F, Costabel U, Poletti V (2014). Interstitial lung disease. Eur Respir Rev.

[2] American Thoracic S, European Respiratory S. American Thoracic Society/European Respiratory Society International Multidisciplinary Consensus Classification of the Idiopathic Interstitial Pneumonias. This joint statement of the American Thoracic Society (ATS), and the European Respiratory Society (ERS) was adopted by the ATS board of directors, June 2001 and by the ERS Executive Committee, June 2001. Am J Respir Crit Care Med. 2002;165(2):277–304. 10.1164/ajrccm.165.2.ats01 11790668

[3] Duchemann B, Annesi-Maesano I, Jacobe de Naurois C, Sanyal S, Brillet PY, Brauner M, et al. Prevalence and incidence of interstitial lung diseases in a multi-ethnic county of Greater Paris. Eur Respir J. 2017. 10.1183/13993003.02419-2016. 10.1183/13993003.02419-2016 28775045

[4] Kim EJ, Collard HR, King TE (2009). Rheumatoid arthritis-associated interstitial lung disease: the relevance of histopathologic and radiographic pattern. Chest.

[5] Winstone TA, Assayag D, Wilcox PG, Dunne JV, Hague CJ, Leipsic J (2014). Predictors of mortality and progression in scleroderma-associated interstitial lung disease: a systematic review. Chest.

[6] Wynn TA (2007). Common and unique mechanisms regulate fibrosis in various fibroproliferative diseases. J Clin Invest.

[7] Wynn TA (2011). Integrating mechanisms of pulmonary fibrosis. J Exp Med.

[8] Meiners S, Eickelberg O, Konigshoff M (2015). Hallmarks of the ageing lung. Eur Respir J.

[9] Korfei M, Ruppert C, Mahavadi P, Henneke I, Markart P, Koch M (2008). Epithelial endoplasmic reticulum stress and apoptosis in sporadic idiopathic pulmonary fibrosis. Am J Respir Crit Care Med.

[10] Castelino FV, Varga J (2010). Interstitial lung disease in connective tissue diseases: evolving concepts of pathogenesis and management. Arthritis Res Ther.

[11] Fischer A, du Bois R (2012). Interstitial lung disease in connective tissue disorders. Lancet.

[12] Raghu G, Anstrom KJ, King TE, Lasky JA, Martinez FJ, Clinical IPF (2012). Prednisone, azathioprine, and n-acetylcysteine for pulmonary fibrosis. New Engl J Med.

[13] Macagno F, Varone F, Leone PM, Mari PV, Panico L, Berardini L (2017). New treatment directions for IPF: current status of ongoing and upcoming clinical trials. Expert Rev Respir Med.

[14] Richeldi L, du Bois RM, Raghu G, Azuma A, Brown KK, Costabel U (2014). Efficacy and safety of nintedanib in idiopathic pulmonary fibrosis. N Engl J Med.

[15] King TE, Bradford WZ, Castro-Bernardini S, Fagan EA, Glaspole I, Glassberg MK (2014). A phase 3 trial of pirfenidone in patients with idiopathic pulmonary fibrosis. N Engl J Med.

[16] Canestaro WJ, Forrester SH, Raghu G, Ho L, Devine BE (2016). Drug Treatment of Idiopathic Pulmonary Fibrosis: Systematic Review and Network Meta-Analysis. Chest.

[17] Torrisi SE, Kahn N, Walscher J, Sarmand N, Polke M, Lars K (2019). Possible value of antifibrotic drugs in patients with progressive fibrosing non-IPF interstitial lung diseases. BMC Pulm Med.

[18] Heukels P, Moor CC, von der Thusen JH, Wijsenbeek MS, Kool M (2019). Inflammation and immunity in IPF pathogenesis and treatment. Respir Med.

[19] Ubieta K, Thomas MJ, Wollin L (2021). The effect of nintedanib on T-Cell activation, subsets and functions. Drug Des Devel Ther.

[20] Bryson T, Sundaram B, Khanna D, Kazerooni EA (2014). Connective tissue disease-associated interstitial pneumonia and idiopathic interstitial pneumonia: similarity and difference. Semin Ultrasound CT MR.

[21] Martinez FJ, Flaherty KR (2017). Comprehensive and individualized patient care in idiopathic pulmonary fibrosis: refining approaches to diagnosis, prognosis, and treatment. Chest.

[22] Hodnett PA, Naidich DP (2013). Fibrosing interstitial lung disease. A practical high-resolution computed tomography-based approach to diagnosis and management and a review of the literature. Am J Respir Crit Care Med.

[23] Wells AU, Kokosi MA (2016). Subclinical interstitial lung abnormalities: toward the early detection of idiopathic pulmonary fibrosis?. Am J Respir Crit Care Med.

[24] Boots AW, van Berkel JJ, Dallinga JW, Smolinska A, Wouters EF, van Schooten FJ (2012). The versatile use of exhaled volatile organic compounds in human health and disease. J Breath Res.

[25] Boots AW, Bos LD, van der Schee MP, van Schooten FJ, Sterk PJ (2015). Exhaled Molecular Fingerprinting in Diagnosis and Monitoring: Validating Volatile Promises. Trends Mol Med.

[26] Van Berkel JJ, Dallinga JW, Moller GM, Godschalk RW, Moonen E, Wouters EF (2008). Development of accurate classification method based on the analysis of volatile organic compounds from human exhaled air. J Chromatogr B Analyt Technol Biomed Life Sci.

[27] Smolinska A, Hauschild AC, Fijten RR, Dallinga JW, Baumbach J, van Schooten FJ (2014). Current breathomics–a review on data pre-processing techniques and machine learning in metabolomics breath analysis. J Breath Res.

[28] Smolinska A, Klaassen EM, Dallinga JW, van de Kant KD, Jobsis Q, Moonen EJ (2014). Profiling of volatile organic compounds in exhaled breath as a strategy to find early predictive signatures of asthma in children. PLoS ONE.

[29] Dallinga JW, Robroeks CM, van Berkel JJ, Moonen EJ, Godschalk RW, Jobsis Q (2010). Volatile organic compounds in exhaled breath as a diagnostic tool for asthma in children. Clin Exp Allergy.

[30] Schnabel R, Fijten R, Smolinska A, Dallinga J, Boumans ML, Stobberingh E (2015). Analysis of volatile organic compounds in exhaled breath to diagnose ventilator-associated pneumonia. Sci Rep.

[31] Van Berkel JJ, Dallinga JW, Moller GM, Godschalk RW, Moonen EJ, Wouters EF (2010). A profile of volatile organic compounds in breath discriminates COPD patients from controls. Respir Med.

[32] Aletaha D, Neogi T, Silman AJ, Funovits J, Felson DT, Bingham CO (2010). 2010 Rheumatoid arthritis classification criteria: an American College of Rheumatology/European League Against Rheumatism collaborative initiative. Arthritis Rheum.

[33] Shiboski CH, Shiboski SC, Seror R, Criswell LA, Labetoulle M, Lietman TM (2017). 2016 American College of Rheumatology/European League Against Rheumatism Classification Criteria for Primary Sjogren's Syndrome: a consensus and data-driven methodology involving three international patient cohorts. Arthritis Rheumatol.

[34] Raghu G, Remy-Jardin M, Myers JL, Richeldi L, Ryerson CJ, Lederer DJ (2018). Diagnosis of idiopathic pulmonary fibrosis. An official ATS/ERS/JRS/ALAT clinical practice guideline. Am J Respir Crit Care Med.

[35] Graham BL, Brusasco V, Burgos F, Cooper BG, Jensen R, Kendrick A (2017). ERS/ATS standards for single-breath carbon monoxide uptake in the lung. Eur Respir J.

[36] Pellegrino R, Viegi G, Brusasco V, Crapo RO, Burgos F, Casaburi R (2005). Interpretative strategies for lung function tests. Eur Respir J.

[37] ATS Committee on Proficiency Standards for Clinical Pulmonary Function Laboratories. ATS statement: guidelines for the six-minute walk test. Am J Respir Crit Care Med. 2002;166(1):111–7. 10.1164/ajrccm.166.1.at1102 12091180

[38] Enright PL, Sherrill DL (1998). Reference equations for the six-minute walk in healthy adults. Am J Respir Crit Care Med.

[39] Box G (1988). Signal-to-Noise Ratios, Performance Criteria, and Transformations. Technometrics.

[40] Walczak B, Massart DL. Wavelets in chemistry. Data handling in science and technology. 22: Elsevier Science; 2000. p. 165–76. 10.1016/S0922-3487(00)80032-5

[41] Eilers PH (2003). A perfect smoother. Anal Chem.

[42] Dieterle F, Ross A, Schlotterbeck G, Senn H (2006). Probabilistic quotient normalization as robust method to account for dilution of complex biological mixtures. Application in 1H NMR metabonomics. Anal Chem.

[43] Breiman L (2001). Random forests. Mach Learn.

[44] Blanchet L, Smolinska A, Baranska A, Tigchelaar E, Swertz M, Zhernakova A (2017). Factors that influence the volatile organic compound content in human breath. J Breath Res.

[45] Engel J, Blanchet L, Bloemen B, van den Heuvel LP, Engelke UH, Wevers RA (2015). Regularized MANOVA (rMANOVA) in untargeted metabolomics. Anal Chim Acta.

[46] Thompson B (2005). Canonical correlation analysis. Encyclopedia of statistics in behavioral sciences.

[47] Benjamini Y, Hochberg Y (1995). Controlling the False Discovery Rate - a Practical and Powerful Approach to Multiple Testing. J Roy Stat Soc B Met.

[48] Smolinska A, Posma JM, Blanchet L, Ampt KA, Attali A, Tuinstra T (2012). Simultaneous analysis of plasma and CSF by NMR and hierarchical models fusion. Anal Bioanal Chem.

[49] Guiot J, Moermans C, Henket M, Corhay JL, Louis R (2017). Blood Biomarkers in Idiopathic Pulmonary Fibrosis. Lung.

[50] Hecker L, Vittal R, Jones T, Jagirdar R, Luckhardt TR, Horowitz JC (2009). NADPH oxidase-4 mediates myofibroblast activation and fibrogenic responses to lung injury. Nat Med.

[51] Agarwal R, Zaidi SI, Athar M, Bickers DR, Mukhtar H (1992). Photodynamic effects of chloroaluminum phthalocyanine tetrasulfonate are mediated by singlet oxygen: in vivo and in vitro studies utilizing hepatic microsomes as a model membrane source. Arch Biochem Biophys.

[52] Adams TB, Cohen SM, Doull J, Feron VJ, Goodman JI, Marnett LJ (2004). The FEMA GRAS assessment of cinnamyl derivatives used as flavor ingredients. Food Chem Toxicol.

[53] Andersen A (2006). Final report on the safety assessment of benzaldehyde. Int J Toxicol.

[54] Lin Z, Li H, Luo H, Zhang Y, Luo W (2011). Benzylamine and methylamine, substrates of semicarbazide-sensitive amine oxidase, attenuate inflammatory response induced by lipopolysaccharide. Int Immunopharmacol.

[55] Singh B, Tschernig T, van Griensven M, Fieguth A, Pabst R (2003). Expression of vascular adhesion protein-1 in normal and inflamed mice lungs and normal human lungs. Virchows Arch.

[56] Jarnicki AG, Schilter H, Liu G, Wheeldon K, Essilfie AT, Foot JS (2016). The inhibitor of semicarbazide-sensitive amine oxidase, PXS-4728A, ameliorates key features of chronic obstructive pulmonary disease in a mouse model. Br J Pharmacol.

[57] Marttila-Ichihara F, Elima K, Auvinen K, Veres TZ, Rantakari P, Weston C (2017). Amine oxidase activity regulates the development of pulmonary fibrosis. FASEB J.

[58] Deetz JS, Luehr CA, Vallee BL (1984). Human liver alcohol dehydrogenase isozymes: reduction of aldehydes and ketones. Biochemistry.

[59] Kneepkens CM, Ferreira C, Lepage G, Roy CC (1992). The hydrocarbon breath test in the study of lipid peroxidation: principles and practice. Clin Invest Med.

[60] Kneepkens CM, Lepage G, Roy CC (1994). The potential of the hydrocarbon breath test as a measure of lipid peroxidation. Free Radic Biol Med.

[61] Phillips M, Altorki N, Austin JH, Cameron RB, Cataneo RN, Kloss R (2008). Detection of lung cancer using weighted digital analysis of breath biomarkers. Clin Chim Acta.

[62] Phillips M, Cataneo RN, Condos R, Ring Erickson GA, Greenberg J, La Bombardi V (2007). Volatile biomarkers of pulmonary tuberculosis in the breath. Tuberculosis (Edinb).

[63] Guan XL, Wu PF, Wang S, Zhang JJ, Shen ZC, Luo H (2017). Dimethyl sulfide protects against oxidative stress and extends lifespan via a methionine sulfoxide reductase A-dependent catalytic mechanism. Aging Cell.

[64] Ostrovsky YuM (1986). Endogenous ethanol–its metabolic, behavioral and biomedical significance. Alcohol.

[65] Bast A, Weseler AR, Haenen GR, den Hartog GJ (2010). Oxidative stress and antioxidants in interstitial lung disease. Curr Opin Pulm Med.

[66] Bos LD, Meinardi S, Blake D, Whiteson K (2016). Bacteria in the airways of patients with cystic fibrosis are genetically cap. J Breath Res.

[67] Gupta S, Shariff M, Chaturvedi G, Sharma A, Goel N, Yadav M (2021). Comparative analysis of the alveolar microbiome in COPD, ECOPD, Sarcoidosis, and ILD patients to identify respiratory illnesses specific microbial signatures. Sci Rep.

[68] Han MK, Zhou Y, Murray S, Tayob N, Noth I, Lama VN (2014). Lung microbiome and disease progression in idiopathic pulmonary fibrosis: an analysis of the COMET study. Lancet Respir Med.

[69] Guilleminault L, Saint-Hilaire A, Favelle O, Caille A, Boissinot E, Henriet AC (2013). Can exhaled nitric oxide differentiate causes of pulmonary fibrosis?. Respir Med.

[70] Malerba M, Radaeli A, Ragnoli B, Airo P, Corradi M, Ponticiello A (2007). Exhaled nitric oxide levels in systemic sclerosis with and without pulmonary involvement. Chest.

[71] Tiev KP, Cabane J, Aubourg F, Kettaneh A, Ziani M, Mouthon L (2007). Severity of scleroderma lung disease is related to alveolar concentration of nitric oxide. Eur Respir J.

[72] Thannickal VJ, Toews GB, White ES, Lynch JP, Martinez FJ (2004). Mechanisms of pulmonary fibrosis. Annu Rev Med.

[73] Bartoli ML, Novelli F, Costa F, Malagrino L, Melosini L, Bacci E (2011). Malondialdehyde in exhaled breath condensate as a marker of oxidative stress in different pulmonary diseases. Mediators Inflamm.

[74] Rihak V, Zatloukal P, Chladkova J, Zimulova A, Havlinova Z, Chladek J (2010). Nitrite in exhaled breath condensate as a marker of nitrossative stress in the airways of patients with asthma, COPD, and idiopathic pulmonary fibrosis. J Clin Lab Anal.

[75] Psathakis K, Mermigkis D, Papatheodorou G, Loukides S, Panagou P, Polychronopoulos V (2006). Exhaled markers of oxidative stress in idiopathic pulmonary fibrosis. Eur J Clin Invest.

[76] Gaugg MT, Engler A, Bregy L, Nussbaumer-Ochsner Y, Eiffert L, Bruderer T (2019). Molecular breath analysis supports altered amino acid metabolism in idiopathic pulmonary fibrosis. Respirology.

[77] Yanagihara T, Kolb M (2019). Molecular breath analysis for IPF: Can we make a few breaths count?. Respirology.

[78] Moor CC, Oppenheimer JC, Nakshbandi G, Aerts J, Brinkman P, Maitland-van der Zee AH, et al. Exhaled breath analysis by use of eNose technology: a novel diagnostic tool for interstitial lung disease. Eur Respir J. 2021; 10.1183/13993003.02042-2020. 10.1183/13993003.02042-2020 32732331

[79] Hayton C, Terrington D, Wilson AM, Chaudhuri N, Leonard C, Fowler SJ (2019). Breath biomarkers in idiopathic pulmonary fibrosis: a systematic review. Respir Res.

[80] Kanoh S, Kobayashi H, Motoyoshi K (2005). Exhaled ethane: an in vivo biomarker of lipid peroxidation in interstitial lung diseases. Chest.

[81] Yamada YI, Yamada G, Otsuka M, Nishikiori H, Ikeda K, Umeda Y (2017). Volatile organic compounds in exhaled breath of idiopathic pulmonary fibrosis for discrimination from healthy subjects. Lung.

[82] Maher TM (2016). Precision medicine in idiopathic pulmonary fibrosis. Qjm-Int J Med.

[83] Guenther A, Krauss E, Tello S, Wagner J, Paul B, Kuhn S (2018). The European IPF registry (eurIPFreg): baseline characteristics and survival of patients with idiopathic pulmonary fibrosis. Respir Res.

